# The use of complementary and alternative medicine by cancer patients

**DOI:** 10.1186/1477-7800-4-10

**Published:** 2007-04-30

**Authors:** Mariama Adams, Andrew Paul Jewell

**Affiliations:** 1 School of Radiography, Faculty of Health and Social Care Sciences, St George's University of London, Cranmer Terrace, London SW17 0RE, UK

## Abstract

The use of Complementary and Alternative Medicine (CAM) among cancer patients is widespread and appears to be increasing. However, it is not clear whether patients use CAM as an 'alternative' to standard oncology care or as an adjunct to the conventional treatment they receive. This study reviews the role of CAM therapies in the management of cancer, from the view of both patients and health professionals and it highlights issues relating to the efficacy of CAM used by cancer patients. Most patients use CAM to 'complement' the conventional therapies of radiotherapy, chemotherapy, hormone therapy and surgery. Health professionals in general have expressed positive views when CAM is used 'complementarily' and not as an 'Alternative'. Results so far published have shown that CAM can contribute to improving the quality of life of cancer patients and their general well-being.

## Background

Many cancer patients have turned to Complementary and Alternative Medicine (CAM) with the hope of finding a cure to their illness as well as to make them feel better. Surveys on the use of CAM by cancer patients have been reported as high as 64% and as low as 7% [1]. As the use of CAM with cancer patients increases, the concern for its efficacy and safety with cancer patients has also increased [2,3]. In spite of the mass use of CAM therapies, very little is known of the efficacy and safety of many of the CAM therapies that cancer patients use.

## Complementary and alternative medicine (CAM)

Complementary and Alternative Medicine (CAM) is defined by the Cochrane collaboration as:" a broad domain of healing resources that encompasses all health system, modalities, and practices and their accompanying theories and beliefs, other than intrinsic to the politically dominant health systems of a particular society or culture in a given historical period" [4]. However, the National Centre for Complementary and Alternative Medicine (NCCAM 2006) in America defines CAM as "a group of diverse medical and health care systems, practices and products that are not presently considered to be part of conventional medicine" [5]. The definition given by Cochrane emphasises healing resources together with its beliefs and theories, while NCCAM talks about systems, practices and products outside conventional medicine. A more recent definition of CAM adapted by the Cochrane School of Complementary medicine is: " diagnosis, treatment and/or prevention which complements main stream medicine by contributing to a common whole, by satisfying a demand not met by orthodox methods or by diversifying the conceptual framework of medicine". Ernst and Cassileth favour this definition because it sees CAM as "complementary" to conventional medicine [1]. The World Health Organisation (WHO) defines CAM as: "A comprehensive term used to refer to both traditional medical systems such as traditional Chinese medicine, Indian ayurverda, Arabic unani medicine, and to various forms of indigenous medicine" [6].

The term Complementary and Alternative Medicine (CAM) is an umbrella term covering both 'complementary therapies' and 'alternative medicines'. Though the phrases are sometimes used synonymously, differences exist between the two. The phrase 'complementary therapy' is defined by cancerBacup as "treatments which are given alongside the conventional cancer treatments" [7]. This means it is there to complement the main conventional therapies such as radiotherapy, surgery, hormone treatment and chemotherapy in the case of cancer patients. The phrase 'Alternative medicine' is described as "practices used instead of standard medical treatment" [8]. However, the definition of "Alternative medicine" outlined by World Health Organisation (WHO) encompasses all forms of healthcare provision, which usually lie outside the official health sector. This definition makes no distinction between the terms "Alternative" and "Complementary". Therefore, in the case of cancer management, anything that falls outside radiotherapy, surgery, hormone therapy and chemotherapy could be considered as Alternative medicine. Because of the meaning attached to the phrase "Alternative therapy", most people prefer to use the term "complementary" instead, although the term is still used to differentiate natural medicine from modern medicine [9]. Nonetheless, the term "Alternative medicine" is popular and much preferred in the United States, as most people still believe that it can sometimes replace conventional medicine in cases where conventional medicine has not lived to expectations [10].

Defining what complementary and alternative medicine (CAM) is, has not been without difficulties. One such problem lies in the fact that there is no clear-cut definition of CAM. What is considered as complementary in the UK is in fact conventional in another country. For instance, Lewith explains that herbal medicine and acupuncture are practiced as Complementary therapy in UK and USA whereas they are considered as part of the main conventional medicine in China [11].

According to CancerBacup, CAM can be divided into three different categories. These are psychological and self-help therapies, which help patients, deal with the emotional and psychological aspects of their illness like stress, anxiety and depression. Among these therapies are counselling, relaxation, healing, visualisation, meditation and art therapy and hypnotherapy. The second group of complementary therapies are considered as physical therapies. These therapies use the sense of touch as the main tool and among them are aromatherapy, acupuncture, massage, reflexology and shiatsu. The last group of complementary therapies are those classified as unconventional medicine or drugs, and includes Homeopathy, Herbal medicine, Essiac, and Bach flower remedies.

However, Montbriand in his study on the overview of complementary therapies chosen by cancer patients had a different grouping for complementary therapies, and described the three types of CAM as psychological, physical and spiritual [12]. The psychological therapies involve some kind of distraction strategies to take the mind of patients off the illness with a positive attitude towards life and finally cure. The physical therapies include herbal tea treatment, injection of thyme enzyme for the enhancement of the immune system, diet alteration and megavitamins. Spiritual therapies involve prayer and healing, for example.

It has been argued that Complementary and Alternative Medicine emphasises the healing of both body and mind. According to Herzberg "While scientific medicine focuses on cures of diseases, complementary medicine is concerned with helping us to heal ourselves" [9] Similarly Fulder, considers that complementary therapy emphasises the restoration of health rather than the removal of sickness [10]. Tschudin, points out that attitude is one of the fundamental differences between complementary therapies and orthodox medicine [13]. While orthodox or conventional medicine views symptoms as hostile and treats them accordingly, Tschudin considers that complementary therapies "use a symptom of illness which a person presents merely as a tool, guide or instructor, to discover more basic imbalances in the person's body, mind or spirit".

## The prevalence of CAM use among cancer patients

There has been a steady increase in the use of complementary and alternative medicine among cancer patients for the past decades [14]. Among the early studies to ascertain the level of CAM use among cancer patients, Downer et al, reported that 16% of cancer patients surveyed in two hospitals in London admitted to using CAM. [15]. This figure is similar to an earlier report in which CAM use was reported at 13% in the USA [16]. However, a recent survey of 127 cancer patients in the UK reported that 29% of their sampled population were using some form of CAM. [17]. In a systematic review of surveys on the use of CAM among cancer patients in 13 countries, Ernst and Cassileth reported a range of 7% to 64% of CAM use among the adult cancer population and the average of 31.4% across all the studies [1]. Some of the commonly used CAM included visualisation, herbs, dietary treatment, meditation, relaxation, homeopathy, hypnotherapy and other mega vitamins. The data collection method used in individual studies was either by interviewing the patients or sending out questionnaires or both. Nine out of the twenty-six surveys were conducted by means of interviewing the patients, fifteen were through questionnaire and two of them employed both methods. The prevalence rate among the nine surveys conducted through interview ranged between 7% and 37% with only one recording a rate of 54% of which spiritual healing was part of the treatment specified. The fifteen other surveys conducted by means of questionnaire reported a prevalence rate of between 16% and 64%. A survey in 14 European countries on the use of CAM among patients with haematological cancers showed that 36% of cancer patients in Europe have used one or more forms of CAM modalities[18]. Similarly, a rate of 40% was reported in the USA after a cross-sectional study of 1904 patients who have previously been diagnosed with cancer [19]. A 2002 National Health Interview survey was used in this study. The most popular CAM therapies used were herbal medicine, deep breathing and meditation. An earlier study conducted in the United States produced a similar prevalence rate of 42% [20]. A survey conducted in Canada reported a 43% prevalence rate of CAM use among cancer patients [21]. In New Zealand, a rate of 49% among 200 cancer patients was reported [22]. The most prominent of the therapies were Vitamins (68%) and Antioxidants (54%).

A similar result was found in Japan, that found use among 44.6% of 3,100 cancer patients [23]. However, 96.2% of the patients were using products such as Chinese herbs, mushroom, shark cartilage and vitamins, which would be considered as CAM products in the west. This emphasises the problem with interpreting some of these data, as the reported varying prevalence rate of CAM use among cancer patients across different surveys has been attributed to the lack of consistency in the definition of CAM and its specificity with regard to what can be considered as a CAM modality [24]. For example, Mao et al included prayer as a CAM modality [19], while others like Harris et al [24] and Scott et al [17] did not. Mao et al reported that over 61% of patients in his study relied on prayer as a form of CAM therapy for their cancer [19]. This is in contrast with a report which mentioned meditation and relaxation as the most commonly used CAM modality [2]. However, the exclusion of prayer from the patients' questionnaire could be a factor. In a study in the UK, aromatherapy and relaxation techniques were among the most popular CAM therapies used by cancer patients [11]. This is clearly in contrast to a survey where herbal medicines were reported to be the most commonly used therapy [18].

Despite these inconsistencies, the socio-demographic pattern of CAM use reveals some consistencies across most of the studies conducted on CAM use among cancer patients. The main recurring themes through out most of the studies were that those who were most likely to use CAM were female, married people, higher earners, better educated and those who have used CAM before their diagnoses [25]. In a study to assess the patterns of alternative medicine use by 319 cancer patients in Australia discovered that most of the patients who employed CAM as part of their cancer management were women (55.5%), people who were married (67.2%) and those with private health insurance (55.2%) [26]. This was consistent with the study carried out in Japan, which had women as the highest single users of CAM modalities in their study of 3100 cancer patients [23]. In the study by Downer et al, 52% of the sample population who have admitted using a form of CAM were women, while 64% of the patients using complementary therapy were married [15]. The study conducted by Molassiotis et al on CAM use among patients with haematological malignancies had 76% of the study sample as married, and over half of sample was women as well [18]. These results may reflect the fact that breast cancer patients are the most likely group to use CAM therapies [15,21,24].

## Patients reasons for using CAM

As more cancer patients turned to CAM in their quest to find a cure for their illness or to better their quality of life, the need to understand their views or perceptions of CAM is of interest. Ernst explained that the reasons given by patients for their use of CAM could be grouped into push factors (negative) which pushes patients away from conventional medicine and pull factors (positive) which relates to the positive aspects of CAM which makes it attractive to patients [27]. Among the push factors are dissatisfaction with conventional medicine, the perceived "poor relationship" with some health care professionals, and desperation on the part of patients to get cured [28]. Hope for a positive outcome of a treatment was mentioned as among some of the positive factors in addition to patients hope for a control over their treatment [2]. Ernst also mentions that good rapport between patients and therapist, as well as the ease at which one can access a CAM modality is a determining factor for patients' use of CAM. These reasons reflect those given by patients in various studies [1,15,17]. Prominent among these were the urge to take control of the treatment and to improve their general condition. In a Norwegian study conducted to ascertain the reasons behind cancer patients' use of non-proven complementary therapies, 36% of 104 patients who participated reported actual improvement in their general condition [29].

The perceptions of patients regarding the use of CAM have been at the centre of discussions whether it is used as an 'alternative' to standard oncology treatments of radiotherapy, surgery and chemotherapy or to 'complement' the conventional treatments [30]. Regardless of whether patients use CAM as an 'Alternative' or 'Complementary' to conventional medicine, they perceive it as a very 'natural therapy' and 'harmless'. In a study by Ponholzer et al on the frequent use of complementary medicine by prostate cancer patients, 90% of the patients were reported to have used CAM with the aim to improve their quality of life [31]. This view is supported by Roberts et al [32] as well Kaasa [33]. In a Norwegian survey, it was reported that most patients were using CAM as it might give them strength to go through the conventional therapies and help to relieve their symptoms [29]. Molassiotis et al conducted a descriptive cross-sectional survey on 127 colorectal cancer patients across seven European countries [18]. Over 47% of the patients reported using CAM with the view of increasing the body's ability to fight off the disease while just fewer than 45% of patients believed that CAM could help improve their physical well-being. In a study by Begbie et al, the most common reason for CAM use was a new hope for cure (49%) and preference for 'natural therapy' was about 20% [26]. Psychological distress was mentioned by Ernst and Fugh-Berman [34] and Holland [35] as among the popular reasons for patients using CAM. In a study on CAM use by cancer patients in Wales, patients cited pain relief as the main reason for using CAM. [24].

Despite the fact that more and more cancer patients are turning to CAM modalities for a number of reasons, few patients disclose this to their health care professionals [32,36]. Studies so far conducted by indicated that just about half of the cancer patients who use CAM inform their doctors of such use [15,25]. Patients perceive a lack of interest on the part of health care professionals or their total disapproval of the therapies [37]. The lack of communication about CAM between patients and health professionals limits the opportunity to discuss the potential benefits and risk of the therapies.

## Efficacy and safety

There has been very little in the way of scientific research into Complementary and Alternative Medicine used by cancer patients in spite of the apparent extensive use of CAM. [38,39]. One of the criticisms levelled against CAM is its' lack of "peer-reviewed scientifically conducted research" as pointed out by the American Society of Clinical Oncology. Several factors have been cited for this. Vickers, including lack of funding and insufficient patient numbers for a study [40]. Hilsden and Verhoef explained that evidence pertaining to the effectiveness of CAM is vital in the decision making of government regarding whether it should be administered or not [41]. Clinical trials are needed to help evaluate and ascertain the benefits of CAM. Randomised control trials (RCT) have been used as the golden rule in evaluating the effectiveness of a medical intervention and reliable evidence in the form of systematic reviews and meta-analyses regarding safety and efficacy are also important [42]. Some Health care professionals in the UK are of the view that for CAM to be accepted as part of the services rendered by the NHS, it should be judged by the same yardstick as any conventional medicine [43]. However, there is the opinion that quality of life and feeling of hope among patients should be included [44]. Most CAM modalities are based on beliefs, practices and traditions of a culture and not on scientific knowledge and their potential benefits and effectiveness based on experiences or testimonials of patients. It has been reported in various studies that patients have used multiple CAM modalities in addition to a conventional treatment, and this has made it difficult to determine the potency of each single modality [45,46].

In a systematic review of Randomised control trials on CAM use by breast cancer patients, Ernst et al identified fifteen studies[47]. Fourteen of the studies used CAM as an adjunct to standard oncology care and only one used the CAM as a sole therapy. Different therapies were tested ranging from psychological and psychosocial support, herbal remedies and massage. It was evident in their review that none of the modality proved effective as an alternative to conventional treatment. It was however evident that some therapies did help patients in terms of their adjustment to life, such as massage and spiritual therapy. Smith et al conducted a study on the outcomes of therapeutic massage for 41 hospitalised cancer patients and they reported a positive outcome for the study [48]. They compared the outcomes of therapeutic massage on a group of patients and that of a nurse interaction on a control group. It was observed that pain, distress, anxiety and sleep quality was worse in the control group and concluded that therapeutic massage help to alleviate pain, distress, as well as improving sleeping patterns. Out of 41, 38 (95%) were men, over 89% were Caucasians and were not in employment. Cassileth and Vickers conducted a much larger study of massage for cancer patients with a sample size of 1290 [16]. Majority of the sample were in-patients (74%), the setting was at the cancer centre for the in-patients, and the outpatients were treated in their various homes. Patients reported over 40% improvement in their pain and fatigue and over 50% in their anxiety levels. A study that looked into how 58 cancer patients adjust to illness when treated with and without CAM in addition to conventional treatments found that patients treated by complementary therapy with conventional therapy fared better psychologically as compared to those treated with only conventional therapy [49]. This was supported by other studies which concluded that aromatherapy to some extent helped improve psychological distress among patients [50,51]. Most of the studies on aromatherapy so far conducted have proven the benefits to cancer patients in terms of managing psychological distress and adjustment to life.

However, in cases where CAM has been used solely as an 'alternative' to standard care, the outcome has been very poor. In a recent study on the outcomes of breast cancer patients who used alternative therapies as primary treatment, it was discovered that the sole use of CAM as primary treatment for breast cancer resulted in increased recurrence and death of patients [52]. Some of the therapies that the patients opted out for included herbal therapy, dietary therapy and high-dose vitamins. A total of 33 breast cancer patients' medical records were reviewed. These patients initially refused standard oncology care or delayed their treatment. Some patients developed disease progression; others had local recurrence while the rest died of metastatic disease. In 2001 in Netherlands a patient died of breast cancer after having CAM therapies instead of conventional therapies [53].

## Perceptions of CAM by health professionals

A study on the knowledge and attitude of oncology professionals towards CAM reported that oncology professionals expressed a negative attitude towards alternative therapies as opposed to complementary therapies [54]. This indicated that health professionals by and large are less sceptical when CAM is solely used to complement mainstream oncology treatments rather than it being used as an alternative. However, the use of CAM as an alternative to conventional medicine has resulted in a negative response from health care professional [55]. This reflects a Korean study to access the knowledge base of both Western and Oriental medical doctors in which more than 50% of Western medical doctors were of the view that "scientifically unproven treatments should be discouraged legally". However, only 11% of the Oriental medical doctors where in agreement to this view. Most complementary therapies are unproven and people need to be cautious of web sites claiming to have alternatives cure for cancer [38]. In a study on physicians' attitude towards the use of complementary therapy by cancer patients in Finland, well over 90% of the physicians were of the opinion that CAM could not wholly cure cancer and therefore must not be encouraged [55]. This was evident in a report from the Netherlands when a patient died from breast cancer after being treated exclusively with various types of CAM therapies, which included acupuncture, faith healing, salt therapy and psychic healing at the expense of radiotherapy and chemotherapy [53]. The fear of patients abandoning or delaying their conventional cancer treatments that are proven in favour of unproven CAM is a major concern [57].

Robotin and Penman [58] and Newell [59] pointed out that the gaps in the knowledge base of healthcare professionals on CAM played a role in their decision-making regarding the use of complementary and alternative medicine. Most health care professionals have admitted that they know very little about complementary and alternative cancer therapies [59]. One study confirmed that most physicians get their information regarding CAM from patients [57]. However patients rarely inform their physicians about their CAM use, which therefore limit physicians' knowledge of CAM. [60]. Insufficient or lack of knowledge on the part of health professionals could be a factor for the lack of approval for CAM use and the subsequent negative attitudes and beliefs [61].

In a survey conducted to assess the familiarity of health professionals with CAM, 67% of the 214 participants mentioned hypnotherapy, acupuncture and imaging as the most familiar of the modalities but would rather recommend support groups for their patients for managing cancer pain [62], although studies conducted so far have shown that health professionals know very little about Complementary and Alternative medicine CAM they have shown interest in CAM [62]. Various reasons have been cited for patients' use of CAM by doctors and other health professionals. Some health professionals perceive cancer patients' use of CAM as a way of life [63]. The idea that 'new innovations' have cropped up and most cancer patients use complementary therapies as a way of living and therefore it is only normal to access CAM rather than having thought through its benefits and risks to the individual patients. The thought by some women in some quarters that 'most women' are using essential oils to help cope with their chemotherapy" had made some women turn to aromatherapy to satisfy their curiosity.

Some health professionals believed that their patients did use CAM therapy as a means of changing their way of life. On the issue of the efficacy of CAM therapies, opinions expressed by health professional have been relatively positive. However, the reported efficacies of CAM were related to the alleviation of side effects to help adjust to illness rather than cancer cure (Gilbar et al 2001). In a survey conducted by Ernst et al (1995) to assess the perception of physicians on CAM effectiveness, 46% of the physicians perceive CAM as moderately effective. It was however noted that younger doctors were more likely to be in favour of CAM compared to the older physician. The possible explanation to this could be that younger doctors are more likely to use CAM for themselves or even recommend it to family members as compared to the older ones (Cunningham et al 1998, Downer et al 1994 & Boon et al 2000).

A concern, which is generally shared by most health care professionals, is the fact that some research carried out has reported possible interactions between some commonly prescribed drugs and CAM products (Izzo and Ernst 2001 & Miller 1998). These interactions if any could have serious effects on the treatment of patients and their subsequent recovery. Lack of clear guidelines with regard to referrals and ultimate responsibility for 'bad outcomes' is one of the reasons why health professional distant themselves from CAM therapies and this makes it difficult to determine their stance with CAM. [61].

## Conclusion

Complementary and Alternative Medicine is an increasingly popular option among cancer patients. However, lack of clear definitions about what constitutes CAM makes it difficult to reach clear conclusions about efficacy and safety. Even so, there is no evidence to suggest that CAM can replace conventional treatment, and there is a need for reliable and consistent information to be made available to patients.
